# *Intimin* (*eae*) and virulence membrane protein *pagC* genes are associated with biofilm formation and multidrug resistance in *Escherichia coli* and *Salmonella enterica* isolates from calves with diarrhea

**DOI:** 10.1186/s13104-022-06218-6

**Published:** 2022-10-11

**Authors:** Shaimaa O. Hasson, Hawraa K. Judi, Hawazen H. Salih, Ameer Al-Khaykan, Sousan Akrami, Sahar Sabahi, Morteza Saki, Zahraa A. Al-Rubaie

**Affiliations:** 1 Department of Biotechnology, College of Biotechnology, Al-Qasim Green University, Babylon, Iraq; 2 Department of Anesthesia, Hilla University College, Babylon, Iraq; 3 Genetic Engineering and Biotechnology Institute for Post Graduate Studies, Baghdad University, Baghdad, Iraq; 4 Department of Air Conditions and Refrigeration Techniques, Al-Mustaqbal University College, Babylon, Iraq; 5 Department of Microbiology, Faculty of Medicine, Tehran University of Medical Sciences, Tehran, Iran; 6 Department of Nutritional Sciences, School of Paramedical Sciences, Ahvaz Jundishapur University of Medical Sciences, Ahvaz, Iran; 7 Department of Microbiology, Faculty of Medicine, Ahvaz Jundishapur University of Medical Sciences, Ahvaz, Iran; 8 Infectious Ophthalmologic Research Center, Imam Khomeini Hospital Clinical Research Development Unit, Ahvaz Jundishapur University of Medical Sciences, Ahvaz, Iran; 9 Department of Microbiology, College of Veterinary Medicine, Al-Qasim Green University, Babylon, Iraq

**Keywords:** Biofilm formation, Calves with diarrhea, *Escherichia coli*, Multidrug resistance, *Salmonella enterica*

## Abstract

**Objectives:**

This study aimed to evaluate the association of the *intimin* (*eae*) and *pagC* genes with biofilm formation and multidrug resistance (MDR) phenotype in *Escherichia coli* and *Salmonella enterica* collected from calves with diarrhea.

**Results:**

Fecal samples (n: 150) were collected from calves with diarrhea. Of 150 fecal samples, 122 (81.3%) were culture positive and 115/122 (94.2%) were Gram-negative bacteria. Among them, *E. coli* (n = 64/115, 55.6%) was the most common isolate followed by *S. enterica* (n = 41/115, 35.6%). Also, 10 (8.6%) isolates were other *Enterobacteriaceae* bacteria including *Klebsiella* and *Proteus* species. Eighty-nine isolates (77.4%) from calf diarrhea, including 52 (81.3%) *E. coli* and 37 (90.2%) *S. enterica* were MDR. The *eae* and *pagC* genes were detected in 33 (51.5%) *E. coli* and 28 (68.3%) *S. enterica* isolates, respectively. There was a strong association between these genes and biofilm formation and MDR phenotype (*P*-value = 0.000). All *E. coli* isolates carrying the *eae* gene were biofilm producers and MDR. Also, all *pagC*-positive *S. enterica* isolates were MDR and 25 (89.3%) isolates of them produced biofilm.

**Supplementary Information:**

The online version contains supplementary material available at 10.1186/s13104-022-06218-6.

## Introduction

Diarrheal infections in calves are among the most common diseases in small livestock and cause significant economic productivity losses for livestock producers [1]. *Salmonella* species, *Escherichia coli*, and *Clostridium perfringens* are among the infectious bacteria associated with calf diarrhea [2, 3].

When *E. coli* and *S. enterica* envade the host, they use different virulence factors to cause infection. *E. coli* is responsible for a variety of diseases in humans and animals, including intestinal and extraintestinal infections. The various virulence factors of *E*. *coli* include hemolysins, colicins, toxins, proteases, adhesion like fimbriae, and cell surface hydrophobicity [4]. *Salmonella* also has several factors responsible for its pathogenicity, including the invasion gene (*inv*A), the fimbriae (*fim*A) gene, and the *spv*ABCD system [5]. *Salmonella* is now the most common cause of bacterial gastroenteritis. Mild diarrhea is a major symptom of salmonellosis caused by *Salmonella* [5].

Farm animals such as cattle are known reservoirs for multidrug resistance (MDR) bacteria such as *E. coli* [6]. The MDR phenomenon has increased worldwide and is considered a public health threat. Several recent studies have reported the emergence of multidrug-resistant bacterial pathogens of various origins, necessitating the proper use of antibiotics, routine antimicrobial susceptibility testing to determine the antibiotic of choice, and screening of emerging MDR strains [7, 8].

The phenomenon of antibiotic resistance is often associated with the development of biofilms in bacterial pathogens such as *E. coli* and *S. enterica* [9]. Biofilms are microbial colonies that adhere to biotic or abiotic surfaces and serve as an important site for horizontal gene transfer [9, 10].

Various genes are involved in the process of biofilm production in *E. coli* and *S. enterica* bacteria [11, 12]. However, not all of them have been studied. For example, there is little information on the function of the *pagC* gene, which is widely distributed in *Salmonella* species [13]. Also, previous studies reported an inverse association between the presence of the *intimin* (*eae*) gene and biofilm formation in *E. coli* [14, 15]. Moreover, to the best of our knowledge, there are no studies that have investigated the association between these genes and the MDR phenotype in bacteria.

Hence, the current study aimed to evaluate the association of the *intimin* (*eae*) and *pagC* genes with biofilm formation and MDR phenotype in *E. coli* and *S. enterica* collected from calves with diarrhea.

## Main text

## Materials and methods

### Bacterial isolation and identification

From October 2020 to January 2021, fecal samples (n: 150) were aseptically collected from calves with diarrhea at Veterinary Teaching Hospital and private clinics in Babylon province in a sterile cup tube and immediately transported to the microbiology laboratory at Al-Qasim Green University. After overnight enrichment in nutrient broth (Merck, Darmstadt, Germany), samples were plated on MacConkey agar (Merck, Darmstadt, Germany) and incubated at 37 °C for 24 h. To obtain a pure culture, colonies appearing after 24 h were re-streaked and then preliminarily identified with microscopy and biochemical tests including urea, triple sugar iron (TSI), indole, motility test, lysine iron agar (LIA), methyl red/Voges-Proskauer (MR/VP), and simmons citrate. Biochemical tests were performed using the VITEK® 2 system (bioMérieux, Inc., Durham, NC, USA) according to the manufacturer’s instructions [16].

## Antimicrobial susceptibility testing using VITEK® 2 system

Antibiotic susceptibility testing (AST) was performed using the VITEK® 2 system (bioMérieux, Inc., Durham, NC, USA) according to the manufacturer’s instructions with AST cards for *Enterobacteriaceae* (ASTN280 cards) [17]. This system performed AST based on the broth microdilution method following the Clinical and Laboratory Standards Institute (CLSI) interpretation criteria [18]. These antibiotics include ampicillin, cefazolin, ceftazidime, ceftriaxone, cefepime, imipenem, tobramycin, ciprofloxacin, and gentamycin. Isolates that were resistant to three or more antimicrobial classes were categorized as MDR [19].

## Biofilm formation detection

Biofilm production was assessed using Congo red agar (CRA) method as described previously [20]. Congo red stain (0.8 g/L) (Sigma-Aldrich, St. Louis, MO, USA), 5% sucrose (50 g/L) (Sigma-Aldrich, St. Louis, MO, USA), and agar (10 g/L) (Sigma-Aldrich, St. Louis, MO, USA) were added to brain heart infusion (BHI) broth (Merck, Darmstadt, Germany) to prepare CRA medium [20]. Bacterial isolates were inoculated onto CRA medium and incubated at 37 °C for 24 h. When black colonies with a dry crystalline appearance were present, the isolates were classified as having a strong biofilm formation. A moderately positive biofilm producer was indicated by dark coloration of colonies in the absence of dry crystalline colony morphology. Non-biofilm producers were defined as colonies that remained pink [20]. A laboratory-confirmed biofilm producer strain was used as a positive control.

## Polymerase chain reaction (PCR)

Colony PCR method was used to detect the *eae* and *pagC* genes in *Enterobacteriaceae* bacteria isolated from feces of calves with diarrheal disease. Genomic DNA was extracted from the bacterial isolates using the Genomic DNA Mini Bacteria Kit (Bio Basic, Markham, Canada) according to the manufacturer’s instructions. The purity of the extracted DNA was determined using a Nanodrop instrument (Thermo Fisher Scientific, Waltham, USA) at 260/280 nm [21]. PCR was performed to amplify the *eae* and *pagC* genes (Table 1) using the specific primers (Bioneer, Daejeon, Korea) [22, 23]. PCR master mix for each gene was prepared using the Maxime PCR PreMix kit (iNtRON Bio, South Korea) according to the company’s instructions as follows: DNA template 5–50 ng in 5 µL, forward primer (10 pmol) 1µL, reverse primer (10 pmol) 1µL and PCR water 13 µL, the total volume 20 µL. PCR conditions for each gene were as follows: initial denaturation at 95 °C for 5 min for one replicate, followed by 30 cycles at 95 °C for 30 s (denaturation), annealing at 58 °C for 30 s, extension at 72 °C for 1 min, and final extension at 72 °C for 5 min for one repeat. PCR products were separated by 1% agarose (Sigma-Aldrich, St. Louis, MO, USA) gel electrophoresis.

**Table 1:** Details of the oligonucleotides used for polymerase chain reaction in this study

PCR Primer	Sequence	Product Size (bp)	Reference
*eae* gene for *Escherichia coli*	F: TCAATGCAGTTCCGTTATCAGTT	482	22
	R: GTAAAGTCCGTTACCCCAACCTG		
*pagC* gene for *Salmonella enterica*	F: CGCCTTTTCCGTGGGGTATGC	454	23
	R: GAAGCCGTTTATTTTTGTAGAGGAGATGTT		

### Statistical analysis

Statistical analysis was performed using Statistical Package for the Social Sciences (SPSS) software version 24.0 software (IBM Corporation, Armonk, NY, USA) [24]. All kinds of data can be processed and analyzed with SPSS software. Significant associations were considered as *P*-value ≤ 0.05 using Chi-square test [25].

## Results

### Phenotypic characteristics of the recovered isolates using VITEK® 2 system

Of the 150 fecal samples collected from calves with diarrhea, 122 (81.3%) were culture positive, while 28 (18.6%) samples were negative. On MacConkey agar, 115/122 (94.2%) were Gram-negative bacteria. Using the VITEK® 2 system, *E. coli* (n = 64/115, 55.6%) was the most common isolate with the following characteristics: motility (+), MR (+), VP (-), indole (+), citrate and urea (-), TSI (acid/acid without H_2_S), and LIA (+). *S. enterica* (n = 41/115, 35.6%) with the following characteristics: motility (+), MR (+), VP (-), indole (-), citrate (+), urea (-), TSI (alkaline/acid with H_2_S), and LIA (+) was the second most abundant isolate. The remaining 10 (8.6%) isolates included *Klebsiella* (5, 4.3%) and *Proteus* (5, 4.3%) species.

## Antibiotic resistance rates and MDR phenotype

Imipenem and ciprofloxacin were the most effective antibiotics with a sensitivity rate of 100.0%. Ampicillin and cefazolin with resistance rates of 100.0% were the less effective antibiotics (Table 2). Eighty-nine isolates (77.4%) from calf diarrhea, including 52 (81.3%) *E. coli* and 37 (90.2%) *S. enterica*, were found to be MDR. Of these, more than 80.0% of the isolates were simultaneously resistant to ampicillin, ceftriaxone, ceftazidime, cefepime, and cefazolin.

**Table 2:** Antibiotic resistance rates and multidrug resistance profiles in *Escherichia coli* and *Salmonella enterica* isolates

Antibiotics	*Escherichia coli* n : 64			*Salmonella enterica* n: 41		
	**Resistant**	**Intermediate**	**Susceptible**	**Resistant**	**Intermediate**	**Susceptible**
	**n (%)**			**n (%)**		
**Gentamicin**	7 (10.9)	0 (0.0)	57 (89.1)	2 (4.9)	0 (0.0)	39 (95.1)
**Tobramycin**	3 (4.7)	0 (0.0)	61 (95.3)	0 (0.0)	0 (0.0)	41 (100.0)
**Imipenem**	0 (0.0)	0 (0.0)	64 (100.0)	0 (0.0)	0 (0.0)	41 (100.0)
**Cefazolin**	64 (100.0)	0 (0.0)	0 (0.0)	41 (100.0)	0 (0.0)	0 (0.0)
**Ceftazidime**	61 (95.3)	0 (0.0)	3 (4.7)	41 (100.0)	0 (0.0)	0 (0.0)
**Ceftriaxone**	61 (95.3)	0 (0.0)	3 (4.7)	40 (97.6)	0 (0.0)	1 (2.4)
**Cefepime**	59 (92.2)	0 (0.0)	5 (7.8)	40 (97.6)	0 (0.0)	1 (2.4)
**Ciprofloxacin**	0 (0.0)	0 (0.0)	64 (100.0)	0 (0.0)	0 (0.0)	41 (100.0)
**Ampicillin**	64 (100.0)	0 (0.0)	0 (0.0)	41 (100.0)	0 (0.0)	0 (0.0)
**Multidrug resistance**	52 (81.3%)			37 (90.2)		

## Biofilm formation

**Fig. 1:**
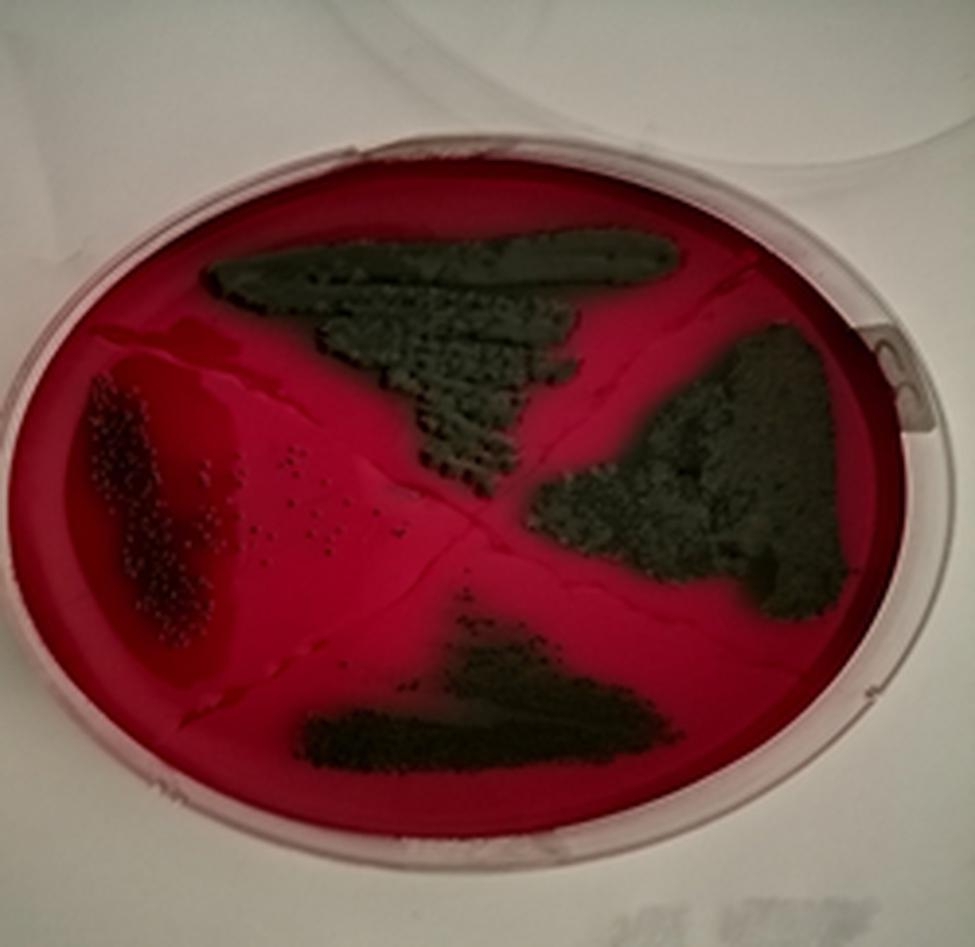
Biofilm formation of *Escherichia coli* and *Salmonella enterica* on Congo red agar

In the current study, all bacterial isolates were evaluated for biofilm formation using the CRA method. The results showed that 40 (62.5%) *E. coli* isolates and 32 (87%) *S. enterica* isolates had the ability to form biofilms (Fig. 1). The black and rough colony on CRA showed the strong biofilm formation activity. All the biofilm producers were MDR isolates. There was a significant association between biofilm formation and MDR phenotype of the isolates (*P*-value = 0.000).

**Association of*eae*and*pagC*genes with biofilm formation and MDR**.

The *eae* and *pagC* genes were detected in 33 (51.5%) *E. coli* and 28 (68.3%) *S. enterica* isolates, respectively (Fig. S1 and S2). There was a strong association between these genes and biofilm formation and MDR phenotype (*P*-value = 0.000). All *E. coli* isolates carrying the *eae* gene were biofilm producers and MDR. Also, all *pagC*-positive *S. enterica* isolates were MDR and 25 (89.3%) isolates of them produced biofilm.

## Discussion

In this study, *E. coli* (64/150, 42.6%) and *S. enterica* (41/150, 27.3%) were the most prevalent bacteria. In a previous study from Egypt, El-Seedy et al. [26] reported a frequency rate of 18.1% and 75.6% for *Salmonella* serovars and *E. coli* in diarrheic calves, respectively. Also, in line with the current findings, Mousa et al. [27] and Shekhar et al. [28] reported a frequency rate of 40.0% and 41.6% for *E. coli* isolates in samples from calves with diarrheal disease, respectively. On contrary, the current result was lower than previous studies by Tadesse et al. [29] and Mohammed et al. [30], who reported prevalence rates of 49%, 85%, and 46.4% for *E. coli*, respectively. This difference in *E. coli* incidence could be due to differences in calf age, study location, sample size, farm size, and hygiene parameters [30].

According to the antimicrobial susceptibility test in this study, the tested strains showed sensitivity to ciprofloxacin, imipenem, tobramycin, and gentamicin with a percentage close to 100%. Meanwhile, resistance to cefazolin, ampicillin, ceftazidime, ceftriaxone, and cefepime exceeded 80%. Similar findings were reported by Mohammed et al. [30] and Manjushree et al. [31]. Eighty-nine isolates from calf diarrhea, including 52 (81.3%) *E. coli* and 37 (90.2%) *S. enterica*, showed the MDR phenomenon. However, Bandyopadhyay et al. [6] reported a lower MDR rate (12.3%) of *E. coli* strains isolated from calves with diarrhea than in the current study. Moreover, in line with the current study, Gebeyehu et al. [32] from Ethiopia reported a 100.0% resistance rate to ampicillin and a 100.0% susceptibility rate to ciprofloxacin among *Salmonella* strains isolated from raw cow milk samples. They also noted a high MDR rate (100.0%) among the isolates [32].

This high prevalence of resistant or multidrug-resistant isolates in the current study may be attributed to the widespread and indiscriminate use of antimicrobials in animals for the treatment, prevention, and control of infectious diseases, and as growth promoters for potential livestock production [32, 33]. Indiscriminate use of these conventional antibiotics without veterinarian prescription and misuse or abuse of veterinary antimicrobials by dairy farmers resulted in the emergence of resistance genes of public concern because they could be transmitted to humans [34]. In recent years, numerous MDR isolates of *E. coli* and *S. enterica* have been reported from several countries [31–33]. Both pathogens utilize multiple antibiotic resistance mechanisms including mobile genetic elements, integrons, plasmids, and efflux pumps [35, 36].

Another mechanism of antibiotic resistance used by some bacteria is biofilm production mediated by several genes [9–12]. In this study, we investigated the association of the *eae* gene of *E. coli* and the *pagC* gene of *S. enterica* with biofilm formation and MDR phenotype to elucidate probable link. To the best of our knowledge, this issue has not been investigated in previous studies from Iraq.

In our study, a strong association was found between the presence of the *eae* and *pagC* genes and biofilm formation and MDR in *E. coli* and *S. enterica* isolates. The *pagC* gene helps *Salmonella* survive macrophage phagosomes [13]. However, in a previous study by Lu et al. [13] from China, deletion of the *pagC* gene was found to promote biofilm formation in *S. enterica* subspecies *enterica* serovar Pullorum. According to their study, deletion of the *pagC* gene reduces the production of outer-membrane vesicles (OMVs) in *Salmonella* isolates, which in turn promotes biofilm stability and bacterial colonization [13]. Moreover, in contrast to this study, Nesse et al. [14] and Stanford et al. [15], claimed an inverse association between the presence of the *intimin* (*eae*) gene and biofilm formation in *E. coli* isolates. Until now, it was not known whether intimin plays a role in biofilm formation in *E. coli* [14]. It seems that more in depth studies are needed to clarify the exact role of the *pagC* and *eae* genes in biofilm formation in *Salmonella* and *Escherichia* species.

In conclusion, this study revealed the association of *eae* and *pagC* genes with biofilm formation and MDR phenomenon in *E. coli* and *S. enterica* species isolated from calves with diarrhea. More in depth molecular based studies are recommended to reveal the detailed mechanisms behind this observations.

## Limitations

No investigation of other related virulence genes and small sample size were the major limitations of this study. Also, the detection of virulence and antimicrobial resistance genes and the correlation between phenotypic and genotypic MDR in the recovered isolates were not be performed.

## Electronic supplementary material

Below is the link to the electronic supplementary material.

Fig. S1 Agarose gel electrophoresis of the PCR products of *Escherichia coli eae* gene (254 bp), M: 100 bp DNA ladder, Lanes 1–9: some positive isolates

Fig. S2 Agarose gel electrophoresis of the PCR products of *Salmonella enterica pagC* gene (212 bp), M: 100 bp DNA ladder, Lanes 1–5: some positive isolates

## Data Availability

The data of the current study are available from the corresponding author on reasonable request.

## Abbreviations

CRA: Congo red agar.
MDR: Multidrug resistance.
PCR: Polymerase chain reaction.
SPSS: Statistical package for the social sciences.
