# Supplementary figures and images for: Intimin (eae) and virulence membrane protein pagC genes are associated with biofilm formation and multidrug resistance in Escherichia coli and Salmonella enterica isolates from calves with diarrhea
Source: BMC Res Notes. 2022 Oct 11;15:321. doi: 10.1186/s13104-022-06218-6 (PMC9552474; doi:10.1186/s13104-022-06218-6)

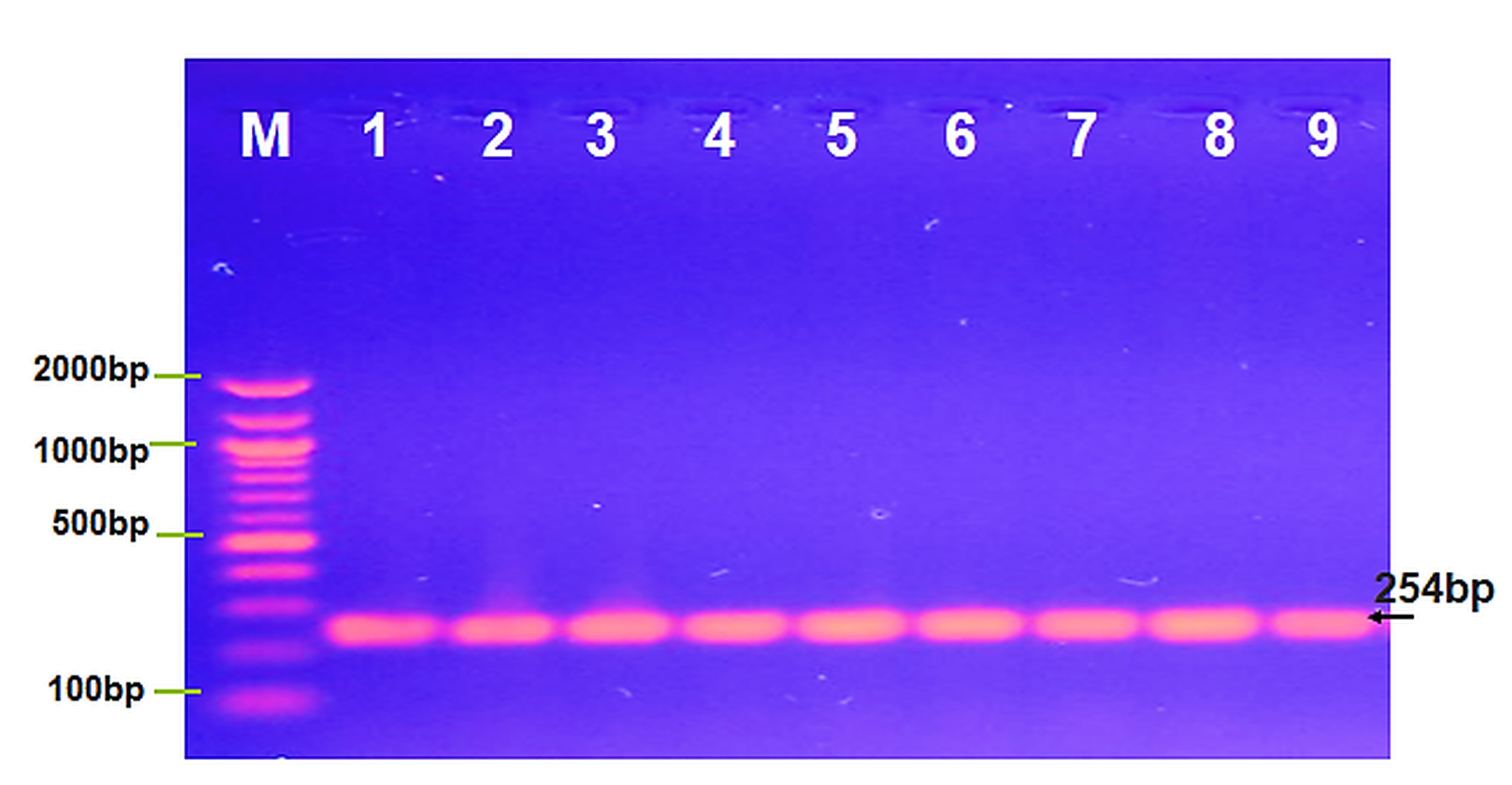

Supplement: Supplementary file 1 — Fig. S1 Agarose gel electrophoresis of the PCR products of Escherichia coli eae gene (254 bp), M: 100 bp DNA ladder, Lanes 1–9: some positive isolates [file 13104_2022_6218_MOESM1_ESM.jpg]

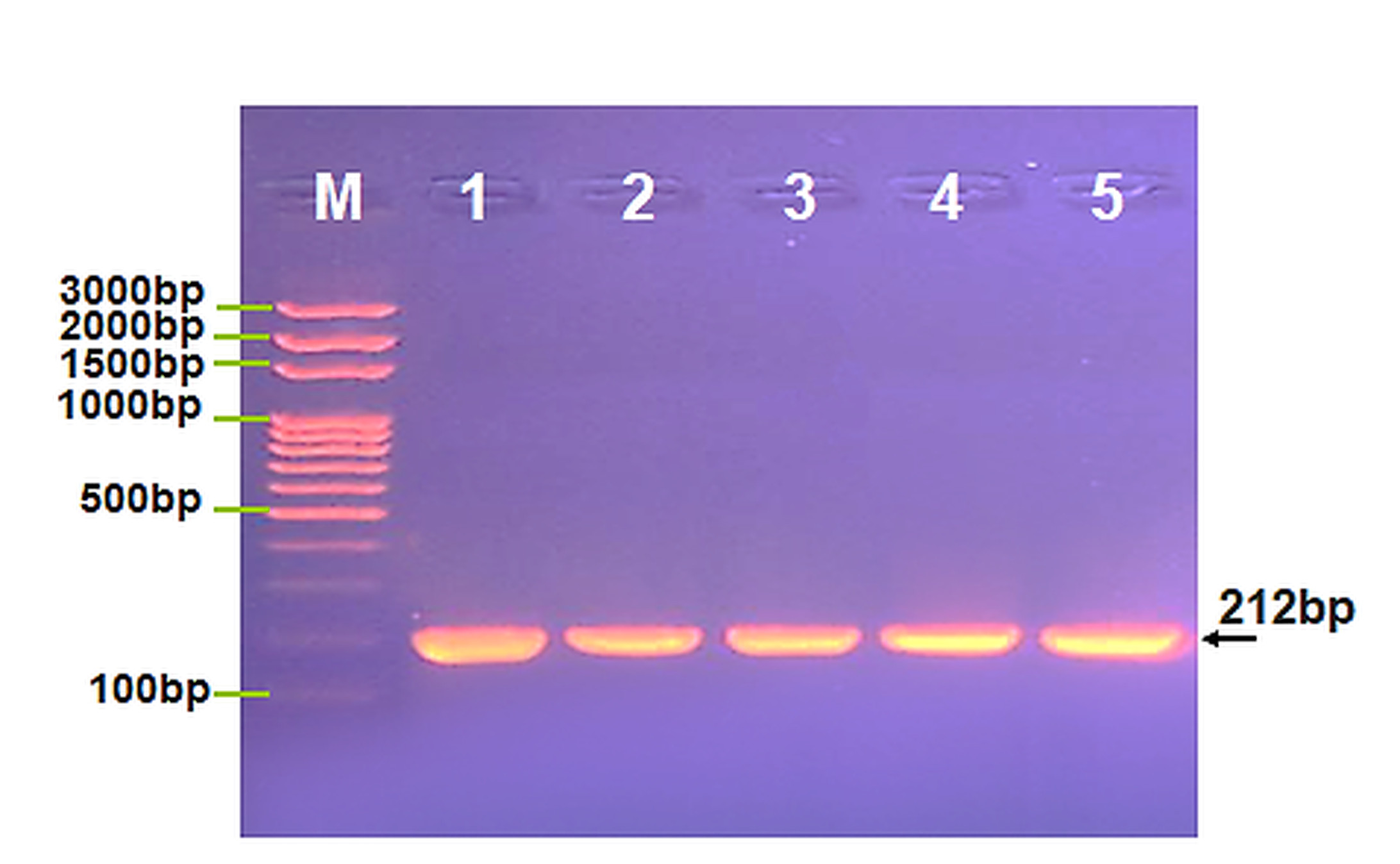

Supplement: Supplementary file 2 — Fig. S2 Agarose gel electrophoresis of the PCR products of Salmonella enterica pagC gene (212 bp), M: 100 bp DNA ladder, Lanes 1–5: some positive isolates [file 13104_2022_6218_MOESM2_ESM.jpg]
